# Role of the trace amine associated receptor 5 (TAAR5) in the sensorimotor functions

**DOI:** 10.1038/s41598-021-02289-w

**Published:** 2021-11-29

**Authors:** D. S. Kalinina, M. A. Ptukha, A. V. Goriainova, N. S. Merkulyeva, A. A. Kozlova, R. Z. Murtazina, T. S. Shemiakova, S. R. Kuvarzin, A. N. Vaganova, A. B. Volnova, R. R. Gainetdinov, P. E. Musienko

**Affiliations:** 1 Institute of Translational Biomedicine, Saint Petersburg State University, Saint Petersburg, 199034 Russia; 2 Sechenov Institute of Evolutionary Physiology and Biochemistry, Russian Academy of Sciences, Saint Petersburg, 194223 Russia; 3 Sirius University of Science and Technology, Department of Neurobiology, Sochi, 354340 Russia; 4 Pavlov Institute of Physiology, Russian Academy of Sciences, Saint Petersburg, 199034 Russia; 5 Children’s Surgery and Orthopedic Clinic, Saint-Petersburg State Research Institute of Phthisiopulmonology, Ministry of Healthcare of the RF, Saint Petersburg, 191036 Russia

**Keywords:** Neurophysiology, Neuroscience, Motor control, Behavioural methods, Electrophysiology, Immunohistochemistry

## Abstract

Classical monoamines are well-known modulators of sensorimotor neural networks. However, the role of trace amines and their receptors in sensorimotor function remains unexplored. Using trace amine-associated receptor 5 knockout (TAAR5-KO) mice, that express beta-galactosidase mapping its localization, we observed TAAR5 expression in the Purkinje cells of the cerebellum and the medial vestibular nucleus, suggesting that TAAR5 might be involved in the vestibular and motor control. Accordingly, in various behavioral tests, TAAR5-KO mice demonstrated lower endurance, but better coordination and balance compared to wild-type controls. Furthermore, we found specific changes in striatal local field potentials and motor cortex electrocorticogram, such as a decrease in delta and an increase in theta oscillations of power spectra, respectively. The obtained data indicate that TAAR5 plays a considerable role in regulation postural stability, muscle force, balance, and motor coordination during active movements, likely via modulation of monoaminergic systems at different levels of sensorimotor control involving critical brain areas such as the brainstem, cerebellum, and forebrain.

## Introduction

Trace amines (TAs) are present in the vertebrate central nervous system (CNS) at very low concentrations that are generally several hundred times lower than those of classical biogenic amines, such as dopamine and serotonin^1,2^. In 2001, two research groups^3,4^ independently reported cloning and identification of a novel family of mammalian aminergic G protein-coupled receptors (GPCRs), which were later re-named as “Trace Amine-Associated Receptors (TAARs)”^5^. There is data indicating that TAs and TAARs are involved in neuromodulation, regulation of metabolism and have different effects on various systems, including CNS^6–9^. However, until recently, all TAARs, except TAAR1, were generally considered only as olfactory receptors involved in the detection of innate odors.

Up to date, the role of TAs and TAARs in the control of sensorimotor functions has been little studied. Several reports have indicated that TAs might affect spinal neural networks responsible for reflex and locomotor activity^10–14^. As for TAARs, TAAR1 is the most studied receptor, but its involvement in the regulation of motor activity has been investigated mostly in terms of interaction with psychostimulant drugs, such as cocaine and amphetamine^15,16^. It has been also reported that the administration of TAAR1 agonist induces the rhythmic locomotor activity of the hindlimb in a spinal animal^13^. TAAR1 mRNA was found in structures involved in the sensorimotor control such as the medulla oblongata, cerebellum, hippocampus, basal ganglia, and spinal cord^3^. The functions of other TAARs have been less studied, but it has been found that several of them, including TAAR5, may also participate in motor control, particularly since the localization of TAAR5 was described in the spinal cord of newborn rats^13^. Our recent study revealed the distribution of TAAR5 in the hippocampus, amygdala, and other limbic structures^17^, along with an increase in exploratory locomotor activity of mice with a knockout of the gene encoding TAAR5 (TAAR5-KO mice). In the present study, we further investigated TAAR5-KO mice in a set of behavioral, electrophysiological, and histochemical experiments to evaluate the role of TAAR5 receptors in various aspects of sensorimotor behaviors.

## Results

### Expression of TAAR5 in the cerebellum and vestibular nuclei

#### LacZ staining in TAAR5-KO mice

**Figure 1:**
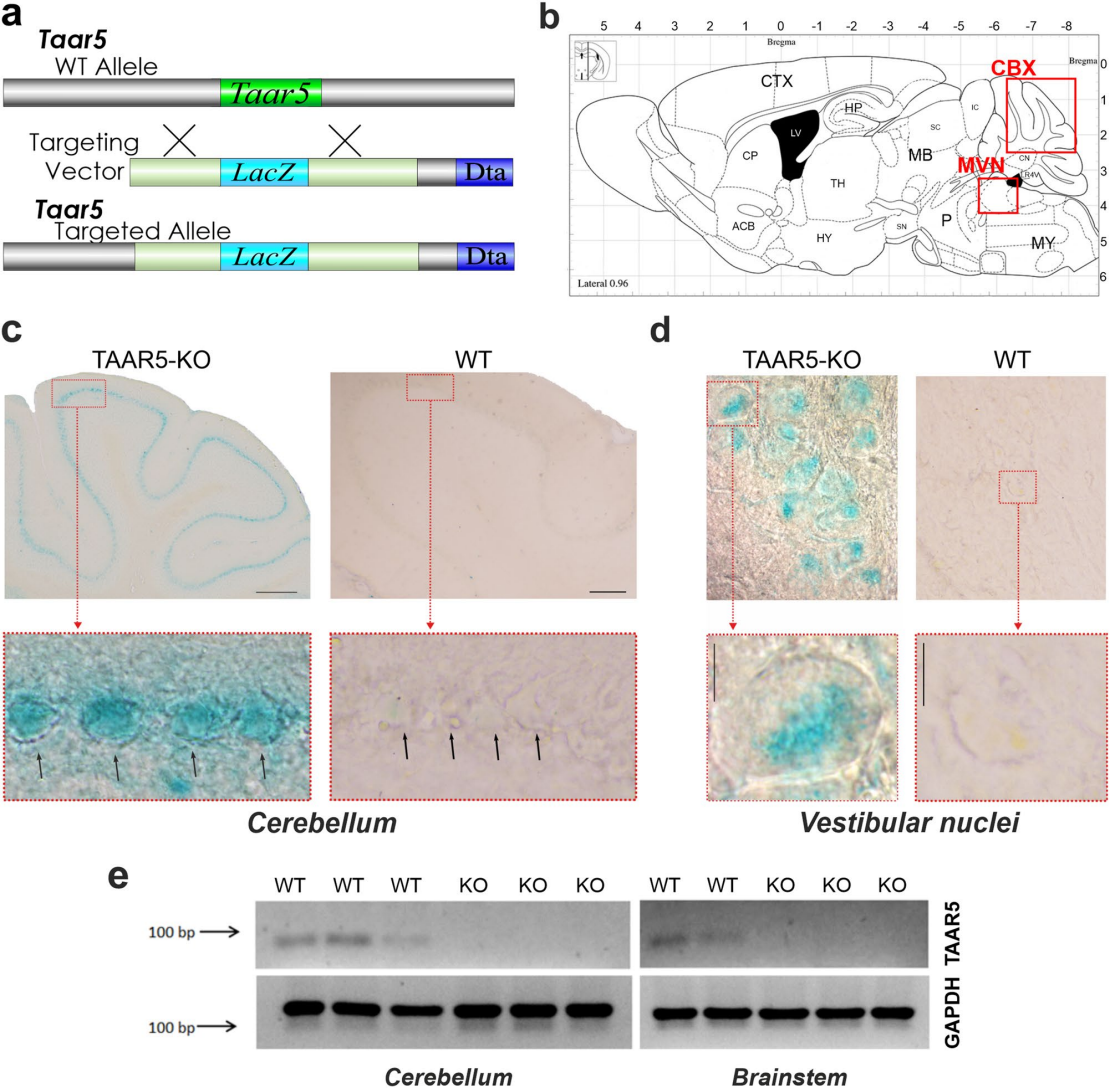
TAAR5 is expressed in the mouse cerebellum and medial vestibular nuclei. (**a**) TAAR5-KO mice were generated using homologous recombination that produces TAAR5 gene inactivation through a replacement vector. The target gene is aligned with the targeting vector containing LacZ-coding sequence^17^; (**b**) a total sagittal view of the mouse brain (modified from Paxinos and Franklin the Mouse Brain in Stereotaxic Coordinates, 200121). (**c**) LacZ stained neuronal profiles of wild-type and TAAR5-KO mice in the Purkinje cell layer of the cerebellum (CBX). Dashed area is an enlarged view of the Purkinje cells. The calibration marker is 100 μm. (**d**) LacZ stained neuronal profiles of wild-type and TAAR5-KO mice in the medial vestibular nuclei (MVN). Dashed area is an enlarged view of the labeled cells. The calibration marker is 10 μm. (**e**) Reverse transcription-polymerase chain reaction (RT-PCR) analysis of TAAR5 mRNA expression in the cerebellum and brainstem of wild-type and TAAR5-KO mice. *ACB* nucleus accumbens, *CBX* cerebellum, *CN* cerebellum nuclei, *CP* caudate-putamen, *CTX* cortex, *HP* the hippocampus, *HY* hypothalamus, *IC* inferior colliculus, *LR4V* lateral recess of 4th ventricle, *LV* lateral ventricle, *MB* midbrain, *MVN* medial vestibular nuclei, *MY* medulla, *P* pons, *SC* superior colliculus, *SN* substantia nigra, *TH* thalamus.

To address the physiological role of TAAR5 in vivo, gene-targeted mice were generated by replacing the bases from 1 to 320 of the *Taar5* gene coding sequence with a *LacZ* (β-galactosidase)-coding sequence^17^ (Fig. 1a). Thus, histochemical staining for LacZ in TAAR5 mutant mice allows analyzing of TAAR5 expression pattern.

First, we detected a discrete and specific LacZ labeling of some motor brain regions (Fig. 1b–d). A clear stain of LacZ marking TAAR5 expression was found within the cerebellum (Fig. 1b,c) in all TAAR5-KO mice analyzed. Bright blue-stained cells having large round soma profiles (382.93 ± 127.1 μm^2^) were documented, well corresponding to the Purkinje cells^18^. LacZ staining in the cerebellum was observed throughout the cerebellar lobules and its pattern does not resemble the striped labeling that of zebrin^19^. Further, we investigated the distribution of LacZ in the brain region related to vestibular control. Densely packed large round or oval-shaped soma cells were observed in the vestibular complex (Fig. 1b,d), mainly in the medial vestibular nucleus (cross-sectional average area of 388.85 ± 49.81 μm^2^). Both shape and size of labeled cells correspond well to vestibular neurons^20^.

#### Transcriptomic analysis of TAAR5 expression in RNA-seq publicly available data

To further validate the expression of TAAR5 in the cerebellum, we performed a transcriptomic analysis of RNA-seq publicly available data. Among the analyzed datasets, three mouse GEO datasets include TAAR5 positive mouse cerebellum samples. In the GSE143671 and GSE83465 datasets, TAAR5 expression is represented in the mouse cerebellum at P2 (in 50% of specimens) and P4 (in all specimens) developmental stages, respectively. TAAR5 is also expressed in all cerebellum samples from P8 mice in the GSE67556 dataset, but in the same dataset, its expression was significantly downregulated at the P60 mice compared to the P8 mice (P adj = 5.809e−11) and TAAR5 mRNA remains detectable only in 50% (12 of 24) of cerebellum samples in this group.

No TAAR5 expression was found in GSE114062 (7 weeks postnatal mice), GSE165105 (15-month postnatal mice), GSE84208 (7 weeks postnatal mice), or GSE79929 (3 weeks postnatal mice) datasets, possibly due to the lower level of TAAR5 expression in adolescent and adult mice compared to early postnatal pups and insufficient RNA-seq depth to demonstrate TAAR5 mRNA in these groups. In fact, in dataset GSE67556, which consists of deeper RNA-seq data compared to other mouse GEO datasets included in the analysis (i.e. GSE67556 it was 200 million reads per sample vs. 30–70 million reads per sample in other datasets), TAAR5 expression was found not only in P8 but also in P60 mice.

The TAAR5 expression pattern analysis in the adult human cerebellum represented in the GSE68559 dataset confirms this consideration. This dataset includes cerebellum samples with significantly different RNA-seq depths ranging from 8.8 to 165 million reads per sample. The expression of TAAR5 mRNA is positive in 4 of 10 samples of the cerebellum in the GSE68559. All TAAR5-positive samples have been sequenced with a depth of 60 million reads or higher. No data were found to perform a similar transcriptomic analysis of expression in the vestibular nuclei.

#### TAAR5 mRNA expression analysis by qPCR in the mouse cerebellum and brainstem

To confirm TAAR5 mRNA expression in the mouse cerebellum and brainstem, RT-qPCR was performed. The specificity of designed primers was verified by the PCR with a plasmid harboring mouse TAAR5 sequence. Its suitability for TAAR5 RT-PCR was confirmed by implementing PCR with cDNA derived from the mouse olfactory epithelium, which is known to express TAAR5, as the template.

Expression analysis of TAAR5 mRNA by qPCR was performed in the cerebellum and brainstem (that includes vestibular nuclei) samples from WT and TAAR5-KO animals (Fig. 1e, Supplementary Fig. 1). Relatively light, but well-defined bands were detected in all cerebellum and brainstem RNA samples from WT animals. Detected bands were noticeably weaker than bands of the housekeeping gene PCR product synthesized on the same cDNA templates. So, the visible difference in the band’s fluorescence intensity seems to be because of the relatively lower TAAR5 expression rather than RNA damage in the course of the sample preparation. The specificity of the detected bands was validated by the lack of corresponding signals in samples from both regions of TAAR5-KO animals.

### Behavioral testing

**Figure 2:**
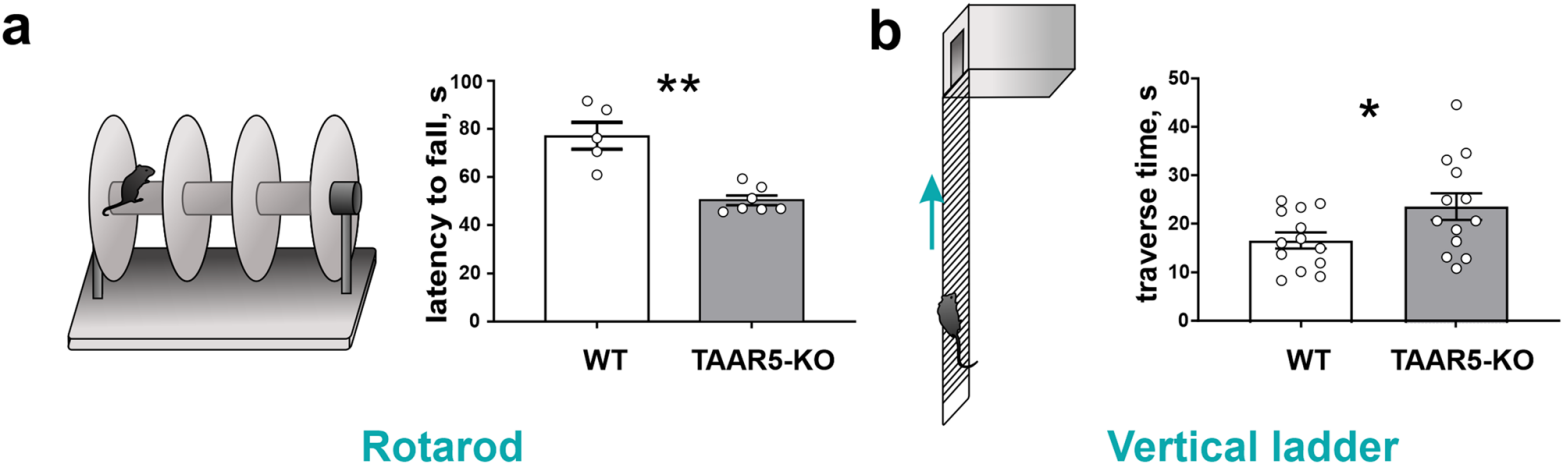
Behavioral testing of motor coordination, endurance, and muscle strength in TAAR5-KO and WT mice. (**a**) Latency to fall from the Rotarod. (**b**) Total time to climb on the Vertical Ladder. Values are presented as mean ± SEM, significance level *P < 0.05, ** P < 0.01.

First, to directly assess motor coordination during a task that required endurance, we used the *Rotarod* test. We performed tests with fast acceleration during the first 30 s, after which max speed was maintained. Knockout mice remained on the rod for a shorter time compared to WT mice (TAAR5-KO: 50.28 ± 2.036 s vs. WT:76.85 ± 5.632 s, Mann–Whitney test, two-tailed, P = 0.0025) while showing similar results during acceleration (all mice were successful in accelerating). The use of acceleration allowed us to separate the results related to motor coordination from those related to endurance. These results hinted at lower muscle strength or endurance in mutant mice (Fig. 2a). To further assess complex motor coordination ability and endurance, we performed several additional tests, including the Vertical ladder test, Static rod test, Vestibular challenge, and Irregular horizontal ladder test.

The *Vertical Ladder* test allows assessment of endurance and muscular strength. Knockout animals climbed slower than the wild-type mice (TAAR5-KO: 23.98 ± 2.756 s vs. WT:17.01 ± 1.621 s, t-test, two-tailed, P = 0.0392, t = 2.181, df = 24) (Fig. 2b). The total number of missteps did not differ between genotypes. These results may indicate that TAAR5-KO mice are less hardy or have lower muscle strength than the wild-type.

**Figure 3:**
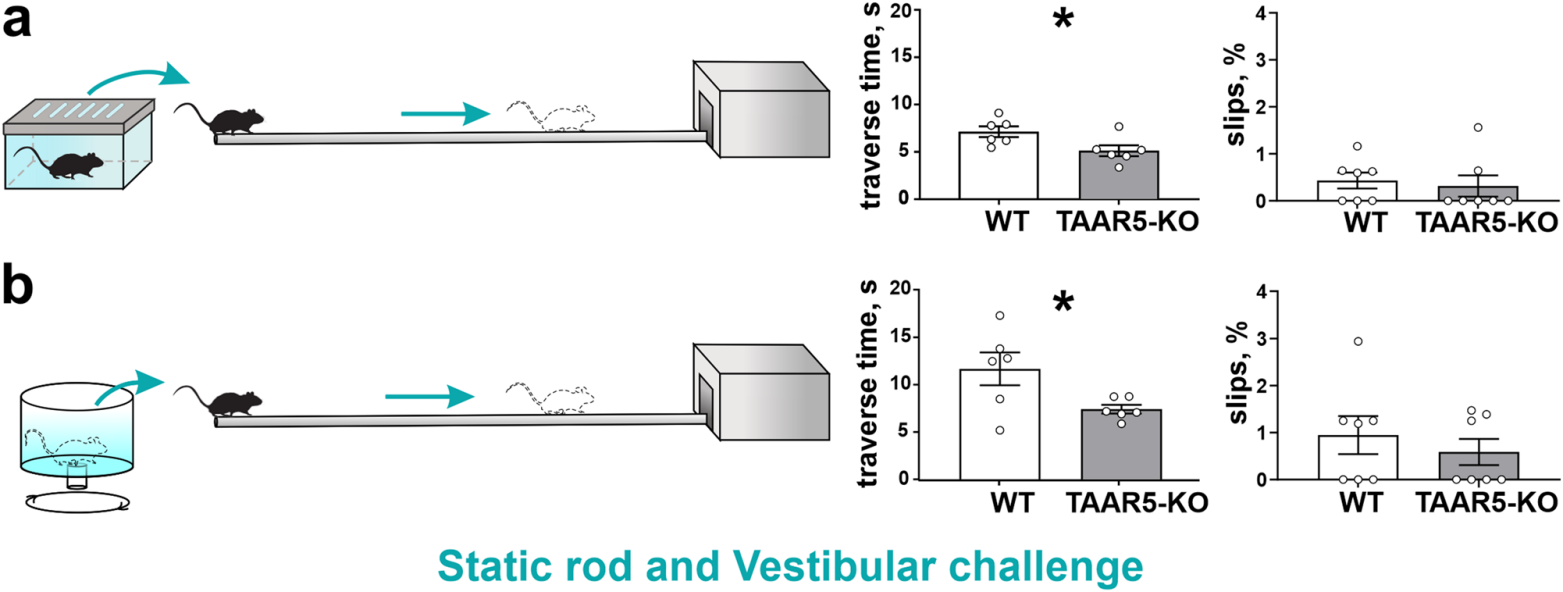
Behavioral testing of motor coordination on the rod, balance, and vestibular control in TAAR5-KO and WT mice. (**a**) Static rod test and traverse time for stepping on it and percentage of slips. (**b**) Vestibular challenge test and traverse time for stepping on it and percentage of slips after 25 s rotation. Values are presented as mean ± SEM, significance level *P < 0.05.

To evaluate motor coordination and balance capacities, we applied the *Static rod* and the *Vestibular challenge tests*^22,23^. Analysis of the percentage of slips did not show any differences between the experimental groups for both tests. At the same time in the Static rod test (Fig. 3a), traverse time was significantly less for TAAR5-KO mice (TAAR5-KO: 5.16 ± 0.59 s vs. WT: 7.19 ± 0.56 s, t-test, two-tailed, P = 0.0451, t = 2.260, df = 11). In the Vestibular challenge test (Fig. 3b), both groups spent more time traversing the rod after rotation, but TAAR5-KO mice were significantly quicker compared to WT controls (TAAR5-KO: 7.42 ± 0.46 s vs. WT: 11.68 ± 1.74 s, t-test, two-tailed, P = 0.0391, t = 2.373, df = 10). Thus, knockout mice demonstrated better abilities at maintaining dynamic balance during locomotion on the Rod.

**Figure 4:**
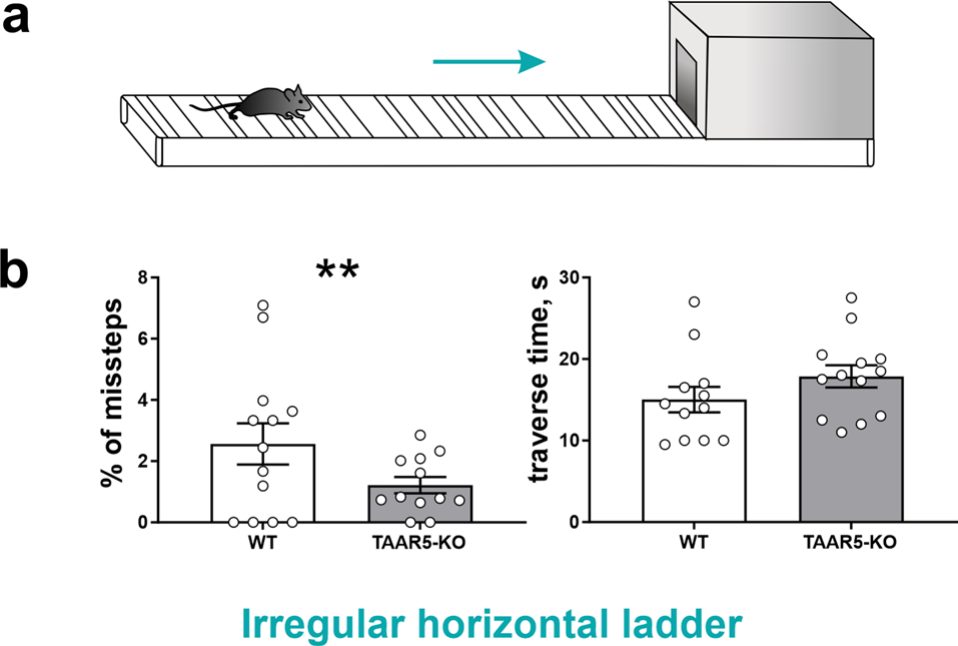
Behavioral testing of complex interlimb coordination required particular attention and concentration in TAAR5-KO and WT mice in the Irregular horizontal ladder test. (**a**) Illustration of the Irregular horizontal ladder test. (**b**) The percentage of missteps and the traverse time. For each animal, the number of missteps was expressed as a percentage of total steps taken by all limbs as they traverse the ladder. Values are presented as mean ± SEM, significance level *P < 0.05, ** P < 0.01.

To assess the dynamic sensorimotor ability in a changeable environment and complex interlimb coordination that requires particular attention and concentration, the *Irregular horizontal ladder test* was used (Fig. 4a,b). Knockout mice had fewer missteps than WT animals (TAAR5-KO: 1.33 ± 0.27% vs. WT: 2.94 ± 0.72%, Chi-square test, two-tailed, Chi-square = 10.02, df = 1, z = 3.166, P = 0.0015), regardless of the limb (fore- or hindlimb, right or left limb). However, the time required to transfer the ladder (Fig. 4b) did not differ (TAAR5-KO: 17.86 ± 1.25 vs. WT: 16.15 ± 1.75, t-test, n.s.). This may indicate better motor coordination in TAAR5-KO mice.

### ECoG and striatal LFP power spectra of TAAR5-KO vs WT mice

**Figure 5:**
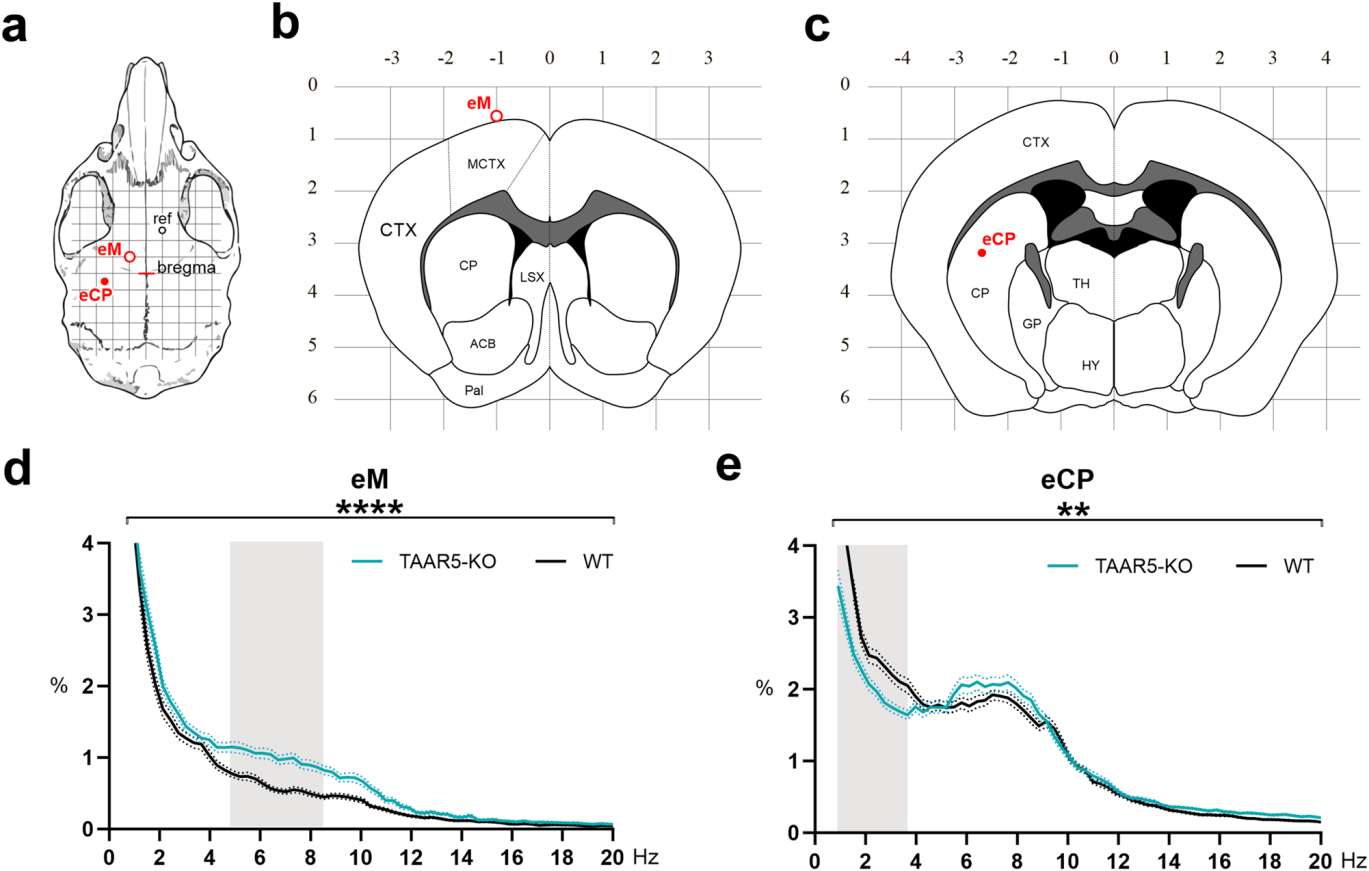
ECoG and striatal LFP. (**a**) Visualization of electrode placement on the skull. Grid 1 mm. (**b**,**c**) Visualization of eM (AP + 1; ML − 1) and eCP (AP − 0.5; ML + 2.5) electrode placements on sagittal slice 1.44 mm lateral to bregma (modified from Paxinos and Franklin the Mouse Brain in Stereotaxic Coordinates, 2001). (**d**) Power spectral density of motor cortex (eM) electrocorticogram. (**e**) Power spectral density of local field potentials in the striatum (eCP). X-axis—signal frequency in Hz; Y-axis—power spectral density in % of total density. Teal and black lines—power spectra of TAAR5-KO and WT mice, respectively; dotted lines—standard error of the mean (SEM). **P < 0.01; ****P < 0.0001 two-way ANOVA of the whole 0.9–20 Hz range. Filled grey area—significant differences in Sidak’s multiple comparisons post hoc test. *eM (circle with red outline)* epidural screw above motor cortex, *eCP (filled red circle)* intracerebral electrode in caudate-putamen, *ref* reference electrode (epidural screw), *ACB* nucleus accumbens, *CP* caudate-putamen, *CTX* cortex, *GP* globus pallidus, *HY* hypothalamus, *LSX* Lateral septal complex, *MCTX* motor cortex, *Pal* pallidum, *TH* thalamus.

Motor cortex electrocorticogram (ECoG) and striatal local field potentials (LFP) were recorded in awake freely moving TAAR5-KO and WT mice (Fig. 5a–c) and analyzed using the Fourier transform. Power spectral density of the whole 0.9–20 Hz range was compared using a two-way ANOVA.

Power spectral density of eM signal (Fig. 5d) was increased in TAAR5-KO mice compared to that of WT mice (two-way ANOVA, group factor F (1, 17,209) = 233.9, P < 0.0001). Sidak’s post hoc multiple comparisons tests showed significant differences in power spectral density between TAAR5-KO mice and WT group in the frequency range of 4.8–8.5 Hz (P < 0.05), which corresponds to the theta band (Fig. 5d, grey area).

Analysis of striatal LFP showed significant differences between TAAR5-KO and WT mice (two-way ANOVA, group factor F(1, 17,292) = 7.340, P = 0.0068) (Fig. 5e). According to Sidak’s post hoc test, significant differences of LFP were found in the frequency range from 0.9 to 3.7 Hz (P < 0.05), which fits in the delta band range (Fig. 5e, grey area).

## Discussion

Classical monoamines (serotonin, dopamine, histamine, etc.) are critical modulators of the brain and spinal cord neural networks that control sensorimotor functions^24^. Trace amines are structurally and functionally close to classical monoamines and can modulate their function by interacting with specific receptors or transporters^25^. However, the role of TAARs, particularly TAAR5, in sensorimotor control remains unknown. In this work, we investigated the sensorimotor functions of mice with a knockout of the gene encoding the receptor TAAR5.

Using LacZ labeling in TAAR5-KO mice, we previously documented the expression of TAAR5 not only in the olfactory system^26^ but also in the limbic brain areas processing olfactory information^17^ and neurogenic zones^27^. In the present study, we extended these observations by detecting LacZ expression also in the distinct motor control centers of the brainstem and cerebellum. Detection of specific signal by qPCR assessment of TAAR5 mRNA in samples from WT but not TAAR5-KO mice further confirmed the expression of TAAR5 in the mouse cerebellum and brainstem. Additionally, transcriptomic analysis of TAAR5 expression in the mouse cerebellum revealed low expression of this receptor in several datasets. Recent detailed transcriptomic analysis of TAAR5 expression in the human brain also showed ubiquitous low TAAR5 expression in the cerebellum and other brain areas including the cortical and limbic brain areas, the amygdala and the hippocampus, the nucleus accumbens, the thalamus, the hypothalamus, the basal ganglia, the substantia nigra, and the white matter^28,29^. Similar conclusions were achieved independently in another transcriptomic analysis study where expression of all TAARs was found in several brain areas including cerebellum and brainstem with TAAR5 appeared to be having the highest expression level^29^. Nevertheless, a relatively low level of expression of TAAR5 in the brain requires further validation by immunohistochemical and other approaches in future studies when and if selective antibodies and tools will become available^7^.

This motivated us to test sensorimotor capacities of TAAR5-KO mice in a variety of behavioral and electrophysiological tests. First, we observed significantly worse performance in the Rotarod test and the Vertical ladder (90°) test in TAAR5-KO mice, which may be the consequence of the decrease in muscle strength or endurance in this group. It has been shown that muscle strength may depend in part on cerebellar function and can be modulated through the cerebellar neural pathways^30^. A likely reason for the sensorimotor characteristics changes observed in TAAR5-KO animals could be the alterations in the functioning of classical monoaminergic systems^17,27^, particularly in histaminergic projections of the cerebellum. This assumption is supported by a recent study, which noted that histamine injection into the structures of the cerebellum in rats leads to an increase in motor coordination and endurance on the Rotarod^31^. Further studies are necessary to evaluate this possibility.

Furthermore, in the Static rod test, TAAR5-KO mice showed a lower traverse time and significantly better motor capacities after rotatory vestibular stimulation compared to control mice. We used a rotatory vestibular stimulus with short duration and low frequency proposed by Tung^32^ to stimulate hair cells in the semicircular canals of the peripheral vestibular labyrinth (predominately the horizontal semicircular canal). The vestibular primary afferent fibers arrive to the central nervous system in the specific regions in the lower pons and upper medulla, known as the vestibular complex. It has been noted that these nuclei also receive other sensory modalities and especially proprioceptive input, and the main tract projecting to vestibular nuclei originates in the cerebellum^33^. The medial vestibular nucleus (MVN) is the largest part of the vestibular complex^34^. This structure processes vestibulo-ocular and vestibulospinal reflexes by the integrating of inputs from vestibular and neck receptors and generating motor output to the oculomotor nuclei and upper cervical cord^35^. Both these brain parts play a key role in the restoration of balance control after vestibular perturbation. Based on the results showing better motor coordination of TAAR5-KO mice in “Static rod test” and “Vestibular challenge” and beta-galactosidase labeling in the MVN, it can be suggested that TAAR5 may be involved in monoaminergic modulation that regulates balance and vestibular control. Dopaminergic projections of MVN have not yet been shown morphologically, but it was found that this nucleus contains meaningful amounts of dopamine^36^. D2 dopamine receptors also have been detected in the MVN^37^, and it has been suggested that histamine and dopamine can modulate MVN excitability. Vestibular nuclei activity is generally considered to be under the control of tonic inhibition regulated by dopamine^35^. In particular, dopamine participates in the regulation of commissural pathways between vestibular nuclei and static reflexes.

At the same time, we found that TAAR5-KO mice have better complex motor coordination (a lower percentage of errors) during walking on the Irregular horizontal ladder, which requires spatial attention. We expect these changes may be associated with the modulation of the serotonergic systems. Recent studies have shown that mice with a knockout gene encoding 5-HT1D serotonin receptors have improved motor skills in the balance beam test^38^. These receptors are localized in the striatum^39^, and the decreased serotonin levels were found in the striatum of TAAR5-KO mice^17^.

Alterations in motor cortex electrocorticogram and striatal local field potentials were recorded in awake freely moving mice lacking TAAR5. Theta-rhythm is widely known to be associated with spatial attention and spatial memory, as well as with specific types of locomotor activity deemed exploratory behavior^40–42^. An increase in power density in the range of this rhythm was observed in the TAAR5-KO mice cortex. Power of theta oscillations is thought to be modulated by serotoninergic projections, both activation of 5-HT2c receptors and serotonin reuptake inhibition leads to suppression of theta rhythm^43,44^. A significant decrease in serotonin level was observed in the hippocampus of TAAR5-KO mice^17^. Such neuromediator imbalance may result in the increased power theta rhythm since theta oscillations recorded above the motor cortex are almost certainly generated by the hippocampus^45^. We also observed a decrease in delta power spectral density in the striatum. Although delta oscillations can frequently be observed during sleep and under anesthesia, they are also known to correlate with several cognitive processes, such as motivation, attention, and concentration^46,47^, which are important for motor coordination and balance ability. Delta rhythm occurs during decision-making and may play a role in suppressing exploratory behavior^46,48^. Oscillations in the 0.5–4 Hz range are also considered to be a robust marker of dopamine depletion, particularly in basal ganglia, and correlate with motor dysfunction^49^. In the case of TAAR5-KO mice, we have previously observed an increase in dopamine tissue levels in the striatum^27^, which could lead to a decrease in the power of delta-range oscillations, as well as to a change in sensorimotor control and improvement of motor coordination in these mice. It is important to note that both the decrease in delta oscillations and the increase in theta oscillations may be indicative of a higher exploratory drive in TAAR5-KO mice. Given that delta oscillations mainly occur when exploration is suppressed, and that theta rhythm, on the contrary, emerges during exploratory behavior, our data suggests increased locomotor/exploratory activity, which has been previously reported^17^ and may play a critical role in complex motor function. At the same time, it should be mentioned that differences in power spectral density can be due to multiple reasons. Not only the decrease in tissue levels of serotonin in the hippocampus and the increase in levels of dopamine in the striatum observed in TAAR5-KO mice may cause altered theta and delta oscillations. Changes in behavioral patterns noted in TAAR5-KO mice^17^ can also potentially contribute to alterations in spectral power, although we attempted to perform experiments when animals were engaged in similar behaviors.

It is known also that TAAR5 is involved in sensing the innate odor-related behavior in mice. In particular, TAAR5-KO mice showed less attraction to a component of male mouse urine trimethylamine^50^ and this effect could potentially result in alterations in motor behavior. For example, it might be expected that wild-type mice may spend more time in tests due to the investigation of “trimethylamine traces”, but in other assays involving an assessment of motor function and exploration (open field test), WT mice showed, in fact, less exploratory behavior compared to TAAR5-KO mice^17^. Furthermore, we noted alterations in several different tests not necessarily involving motor functions and thus we believe that only this fact cannot fully explain the data observed.

As has been pointed out earlier^17^, we confirm here that TAAR5 (and likely TAARs in general) is not just “an olfactory receptor” involved in sensing innate odors^51^ but also participates in neuromodulation of sensorimotor control in the brainstem, cerebellum, and forebrain. Taking into account the specific effects in musculoskeletal systems as well as our previous finding of activation of adult neurogenesis inTAAR5-KO mice^27^, we consider the potential utility of future TAAR5-targeted agents in pharmacotherapy of neuromotor disorders. Further detailed investigations are warranted to evaluate this possibility. Finally, given the close similarity between members of the TAAR family, it would be important to evaluate the role of other TAARs in sensorimotor functions. Intriguingly, the expression of other TAARs (TAAR1, TAAR8) has been previously reported in the cerebellum of mice and humans^27,52^.

## Conclusion

To sum up, we demonstrate here that TAAR5-KO mice have a specific motor phenotype characterized by a decrease in muscle force and an improvement in balance and motor coordination that is related to the distribution of the TAAR5 in vestibular nuclei, cerebellum, as well as altered striatal delta- and cortical theta-oscillations. These results demonstrate the involvement of TAAR5 in the control of sensorimotor activity that can be due to the modulatory effect of TAAR5 on the dopaminergic, serotonergic, and potentially other aminergic systems, which carry out supraspinal regulation of neural networks involved in the control of postural and locomotor functions.

## Methods

### Animals

Behavioral testing and electrophysiological recordings were performed on adult male mice, while histochemical experiments were carried out on adult mice of both genders. The TAAR5-KO mice expressing beta-galactosidase mapping TAAR5 expression were generated by Deltagen Incorporation (San Mateo, CA, USA) and distributed by the NIH Knockout Mouse Project (KOMP). Breeding and genotyping of TAAR5-KO and wild-type (WT) mice were performed as described previously17. All procedures involving animals and their care were approved by the Ethics Committee of St. Petersburg State University, St. Petersburg, Russia (approval number 131-03-4 and 131-03-5 and conducted under Russian legislation according to Good Laboratory Practice standards (directive # 267 from 19.06.2003 of the Ministry of Health of the Russian Federation) and in compliance with ARRIVE guidelines.

The mice were housed three to five per cage before the operating procedure and individually after the procedure. Standard lab conditions were maintained (12 h light/dark cycle, 21 ± 1 °C and 40–70% humidity), with food and water provided ad libitum. All experiments were conducted during the light phase. The same cohort was used in vertical and horizontal ladders while static rod and vestibular challenges were performed in another group, the other tests were performed in separate groups of animals. The total number of animals used in the study: 39 TAAR5-KO and 38 WT (3–6 months old).

### Expression of TAAR5 in the cerebellum and vestibular nuclei

#### LacZ staining

TAAR5 expression was analyzed with the use of the inserted LacZ reporter gene (Fig. 1a). All procedures for the LacZ labeling and visualization were documented previously^17,27^. In brief, animals were pre-anesthetized with isoflurane and anesthetized with 2 g/kg urethane and sacrificed by decapitation (TAAR5-KO n = 5, WT n = 5). Thereafter, the brain was removed from the skull, rinsed in phosphate-buffered saline (PBS) 3 times, and immediately fixed overnight in 4% paraformaldehyde (PFA). Frontal and sagittal sections (40 μm) were prepared using microtome Leica CM-3050S (Wetzlar, Germany). Both free-floating and attached sections were used for LacZ staining. Sections were rinsed in 0.02% NP40 (Sigma, ab142227) and 2 mM magnesium chloride in PBS, and thereafter incubated overnight in the staining solution containing 1 mg/ml X-gal (5-Bromo-4-chloro-3-indolyl β-d-galactopyranoside, Sigma, B4252), 5 mM potassium ferri- (K3Fe(CN)6, Sigma, 244023), 5 mM ferrocyanide (K4Fe(CN)6, Sigma, 455989) and 2 mM MgCl_2_, at + 37 °C. Sections were cover-slipped with DPX (Sigma-Aldrich) and thereafter processed using Olympus BX51 microscope (Olympus Corporation, Japan). LacZ labeled cells were blue stained.

#### Bioinformatics: gene expression analysis

RNA-seq transcriptomic data was retrieved from the GEO (Gene Expression Omnibus) repository^53^. First, the GEO browser available on https://www.ncbi.nlm.nih.gov/geo/browse/ was searched for the term “cerebellum”. Datasets were included in the review if suitable to according inclusion criteria: (1) At least 30 million reads in the SRA file for all samples in the dataset; (2) Availability of dataset on GEO RNA-seq Experiments Interactive Navigator platform (GREIN, available at: http://www.ilincs.org/apps/grein/?gse=)^54^.

After exclusion of all non-appropriate datasets, GSE114062, GSE165105, GSE84208, GSE67556, GSE143671, GSE83465, and GSE79929 mouse cerebellum datasets and GSE68559 human cerebellum datasets were included in the analysis. All RNA-seq datasets were analyzed by the online GREIN platform^54^. TAAR5 expression was considered as positive if at least 10 reads per sample were identified as TAAR5 mRNA fragments (counts), then the data were CPM-normalized and differential gene expression was estimated. Raw P values were adjusted for multiple testing using the Benjamini–Hochberg procedure and only adjusted P (Padj) values higher than 0.05 were considered significant.

#### TAAR5 mRNA expression analysis by qPCR

Tissues from 3 animals of both genotypes were dissected on ice, frozen in liquid nitrogen, and stored at − 80 °C.

RNA isolation from brainstem and cerebellum was performed using GeneJet RNA Purification kit (Thermo Scientific) according to the manufacturer’s instructions. The RNA was eluted in RNase-free water and kept at − 80 °C until used. RNA concentration was quantified using spectrophotometry (NanoDrop, München, Germany). To eliminate any remaining genomic DNA TURBO DNA-free kit (Thermo Scientific) was used on RNA samples. 1 μg of RNA (brainstem) and 0.6 μg (cerebellum) was taken for the synthesis of cDNA using Revertaid Reverse Transcriptase (Thermo Scientific). Briefly, reverse transcriptase and reaction mix containing 1 μM random hexamer primers were added to DNAse‐treated RNA and exposed to the following protocol: annealing at 25 °C for 10 min, transcription at 42 °C for 90 min, and termination at 70 °C for 10 min. cDNA samples were stored at − 20 °C. 1 µl of cDNA was used for qPCR. As a control for the successful removal of genomic DNA, each sample was exposed to the same treatment except that the reverse transcriptase was not added (RT—control). Gene expression was assessed using qPCR. Reactions were performed in duplicates using qPCRmix-HS SYBR (Evrogen, Russia) under the following condition: 95 °C for 5 min followed by 35 cycles of 95 °C for 20 s, 60 °C for 20 s, and 72 °C for 30 s.

Primers were designed to detect mouse TAAR5 (mTAAR5_F1: 5′-5-ttctgctaccaggtgaatgggt-3′, mTAAR5_R1: 5′-gccagatagatgacgacctgga-3′) and were tested for specific target amplification in the sample compared to the RT—control using melting curve analysis (from 55 to 95 °C) and 2% agarose gel electrophoresis. Mouse glyceraldehyde 3-phosphate dehydrogenase (GAPDH) gene (primers: mGAPDH_F2: 5′- ttgatggcaacaatctccac-3′, mGAPDH_R2: 5′-cgtcccgtagacaaaatggt-3′), which is a housekeeping gene was included as an internal control.

### Behavioral testing

*Rotarod* (Fig. 2a) is used to assess motor coordination and balance, as well as endurance of the animal^22,52^. Before testing, animals were pre-trained for three days (24 rpm, 1 min, 3 trials with at least 2 min of rest between trials) to adapt mice to the apparatus and movement on the rotation rod (40 mm diameter, Neurobotics, Russia). Then we performed an accelerating rotarod, i.e., stepwise speed increase from 10 to 30 rpm (every 5 s by 5 rpm), after which a speed of 30 rpm was maintained (the whole test lasted 5 min). Time of fall for each mouse was noted. The Rotarod test was performed on two groups of mice: TAAR5-KO (n = 7), WT (n = 5).

*Vertical ladder* (Fig. 2b) allows to evaluate of muscle strength and endurance of the mouse and is a simpler modification of vertical grid test^55^. The ladder is 50 cm in height, and it has 90° degrees of inclination and metal rungs (3 mm in diameter) with a distance of 1 cm between rungs. A plastic box was located at the top of the ladder. The mouse was placed on the bottom rung, and the time to climb up to the box and the number of missteps on the ladder were noted. The test consisted of 2 trials, with a one-minute intertrial interval. Vertical ladder test was performed on two groups of mice: TAAR5-KO (n = 13), WT (n = 13).

#### Static rod and vestibular challenge

In addition, we evaluated motor coordination and balance capacities of the mice using Static rod and Vestibular challenge tests^22,23^. A 100 cm long rod was placed 80 cm above the floor, with one tip fixed under the goal box (“fixed tip”) and the other one suspended (“suspended tip”). The rod was marked at 10-cm interval from the target box (“finish point”). Mice were placed on the suspended tip with nose oriented towards the goal box. Traverse time (Fig. 3a) was registered (without any disturbance). To assess the possible impairment in vestibular control, the Vestibular challenge test (Fig. 3b) was used^32^. The mouse was placed inside a plexiglass rotating box. A 3 Hz clockwise rotation lasting for 25 s was used to disturb the vestibular system. After such perturbations, the animal was placed on the rod, then traverse time was examined (from the end of the rotation to crossing the "finish point"). For both tests, the number of correct steps and the number of slips for each limb were counted separately, then the results obtained were summed up for all 4 limbs and the slips were expressed as percentage of slips out of the total amount of steps of all limbs. The Static rod and Vestibular challenge tests were performed on two groups of mice: TAAR5-KO (n = 6), WT (n = 6).

*Irregular horizontal ladder* with irregularly located round rungs (Fig. 4a) was used to evaluate complex sensorimotor coordination and intricate combination of fore- and hindlimbs that require particular attention and concentration. Apparatus consisted of stainless-steel rungs (3 mm diameter), which were located between clear polycarbonate walls. The side walls were 50 cm long and 21 cm high measured from the rungs, which were lifted off the floor. The test was repeated 3 times, but every trial had a distinct pattern of rung location to prevent memorization of the order of the rungs. The correct steps and full missteps of all four limbs were counted. The full misstep was defined as a complete fault in which the limb fell between the rungs, causing disturbance of balance and body posture. 15–20 steps were analyzed separately for each limb and the results were presented as percentages of full missteps out of all steps. Irregular horizontal ladder test was performed on two groups of mice: TAAR5-KO (n = 13), WT (n = 12).

All behavioral tests were performed with video recording and analyzed after experiments. All quantitative data obtained are presented as mean ± SEM. Student’s t-test was used for normally distributed data (Shapiro–Wilk normality test), for other data—Mann–Whitney U-test.

### Chronic electrophysiological recordings

#### Surgical procedure

Two types of electrodes were used, epidural screws for electrocorticogram (ECoG) recordings (0.5 mm in diameter; 1 mm in length; steel) and intracerebral electrodes for local field potential (LFP) recordings [50 µm in diameter; 1.2 mm/3.2 mm in length; tungsten wire in perfluoroalkoxy polymer isolation (795500, A-M Systems)]. For each animal, three electrodes were implanted under general anesthesia (200 mg/kg Zoletil intraperitoneally; Xylazine 0.2 mg/kg intramuscularly): epidural reference electrode (2 mm anterior to bregma and 1 mm lateral to the midline); motor cortex (eM) epidural electrode (1 mm anterior to bregma and 1 mm lateral to the midline); striatal (eCP) intracerebral electrode (3.2 mm in length, 0.5 mm posterior to bregma and 2.5 mm lateral to the midline)^21^ (Fig. 5a,b). Electrodes were placed using a micromanipulator in a stereotaxic frame. Electrodes were fixed on the skull with dental cement (Acrodent, JSC Ctoma, Ukraine). The experiments were started three days after the electrode implantation. The series of three experiments were conducted on each animal no more frequently than once every other day.

#### ECoG and LFP recordings

The experimental setting for electrophysiological recordings consisted of an amplifier (× 1000 gain, USF-8; Beta Telecom), analog-to-digital converter L-791 (L-CARD) and Bioactivity Recorder v5.44 software (D.A. Sibarov, Biotechnologies). During the recording process, the animal was placed in a 20 × 20 × 25 cm plexiglass box, which along with the amplifier was located in an isolated grounded setting. Signal was band-pass filtered between 0.1 and 200 Hz, and digitized with 2500 samples per second per channel. The brain activity of TAAR5-KO and WT groups was recorded for 20 min in awake freely moving mice habituated to the experimental cage and displaying no obvious differences in behaviors between genotypes, 3 times for each animal on different days. Only those epochs in which animals exhibited similar behavior were included in the analysis. These recordings were then used to compare spectral characteristics of TAAR5-KO and WT mice electrophysiological activity.

#### Data analysis

Using the Bioactivity Recorder software, 12 epochs without artifacts, 20 s in length, were picked out from each recording, then Fourier transform was performed using Clampfit 10.2.0.16 software (MDS Analytical Technologies). The resulting power spectra (frequency in Hz, power spectral density in μV^2^) of WT mice were compared to those of TAAR5-KO mice. The resulting data was in the 0.9–20 Hz range after data in the 0–0.9 range was excluded due to the abundance of artifacts. The data was analyzed after normalization, which transforms all values from one data set into percentages of the sum of all power spectra values, which was necessary for standardization across the group. All data were tested for Gaussian distribution with the use of the Shapiro–Wilk normality test. All data were analyzed using two-way ANOVA (analysis of variance). Sidak’s multiple comparisons test was performed to distinguish the most prominent differences. All statistical analyses were performed by using GraphPad Prism 9.0.2. (GraphPad Software, Inc., San Diego, CA, USA). All data are presented as mean ± standard error (SEM).

Electrophysiological studies were conducted on two groups of animals: TAAR5-KO (n = 5) and WT (n = 6) mice.

## Supplementary Information

Supplementary Figure 1.
