# Supplementary material for: Role of the trace amine associated receptor 5 (TAAR5) in the sensorimotor functions
Source: Sci Rep. 2021 Nov 29;11:23092. doi: 10.1038/s41598-021-02289-w (PMC8630200; doi:10.1038/s41598-021-02289-w)

Supplementary figure 1. Full-length agarose gel: Reverse transcription-polymerase chain reaction (RT-PCR) with TAAR5 and GAPDH (housekeeping gene) specific primers using RNA isolated from the cerebellum and brainstem of WT and KO animals confirmed TAAR5 mRNA expression in the cerebellum and brainstem and a corresponding decrease in knockout. Red is a source of cropped image.


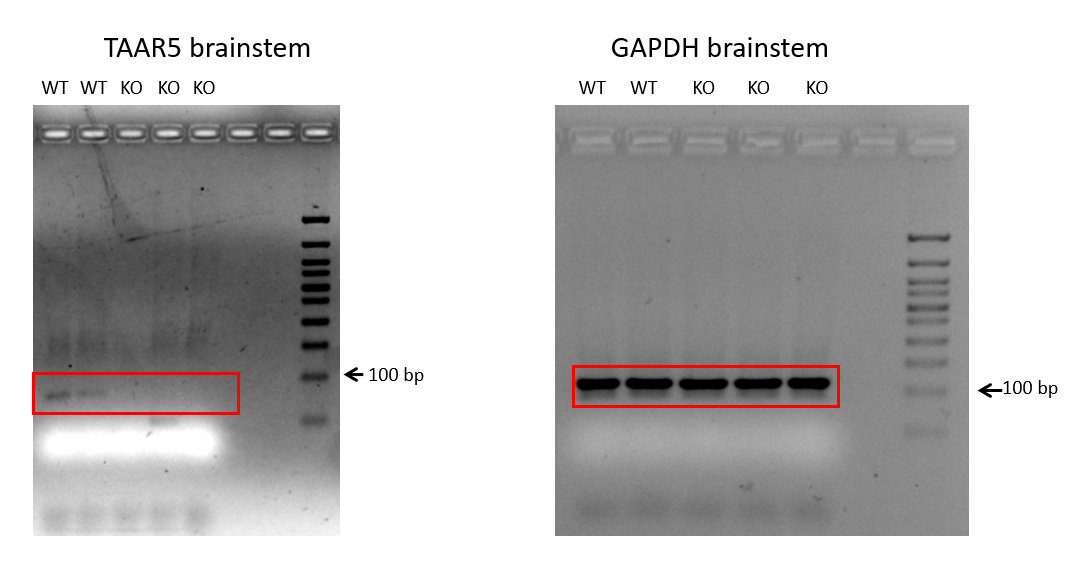

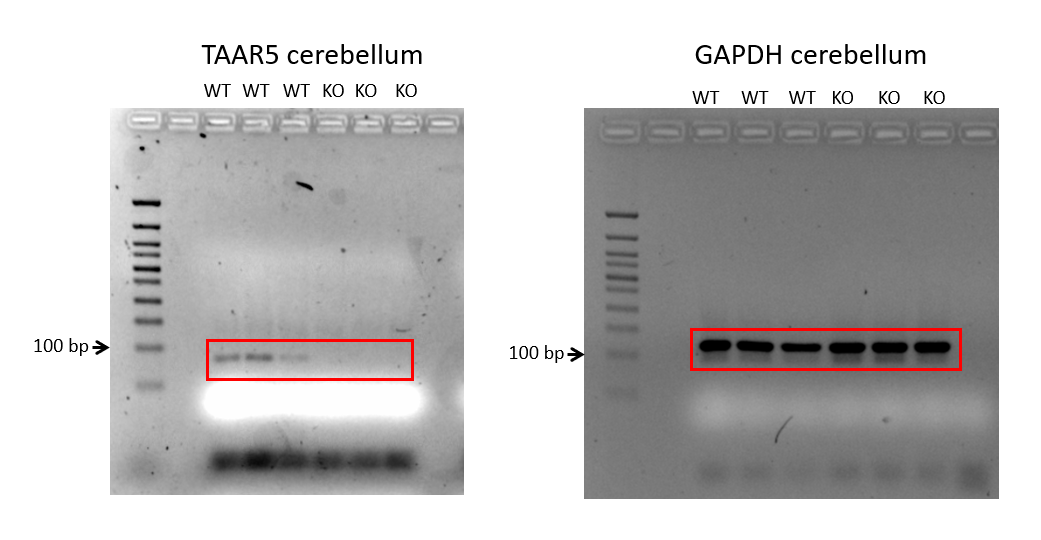

Supplement: Supplementary file 1 — Supplementary Figure 1. [file 41598_2021_2289_MOESM1_ESM.docx]
